# Supplementary material for: Pullulan-Based Active Coating Incorporating Potassium Metabisulfite Maintains Postharvest Quality and Induces Disease Resistance to Soft Rot in Kiwifruit
Source: Foods. 2023 Aug 24;12(17):3197. doi: 10.3390/foods12173197 (PMC10487164; doi:10.3390/foods12173197)
Supplement: Supplementary file 1 [file foods-12-03197-s001.zip › Figure S1.pdf]

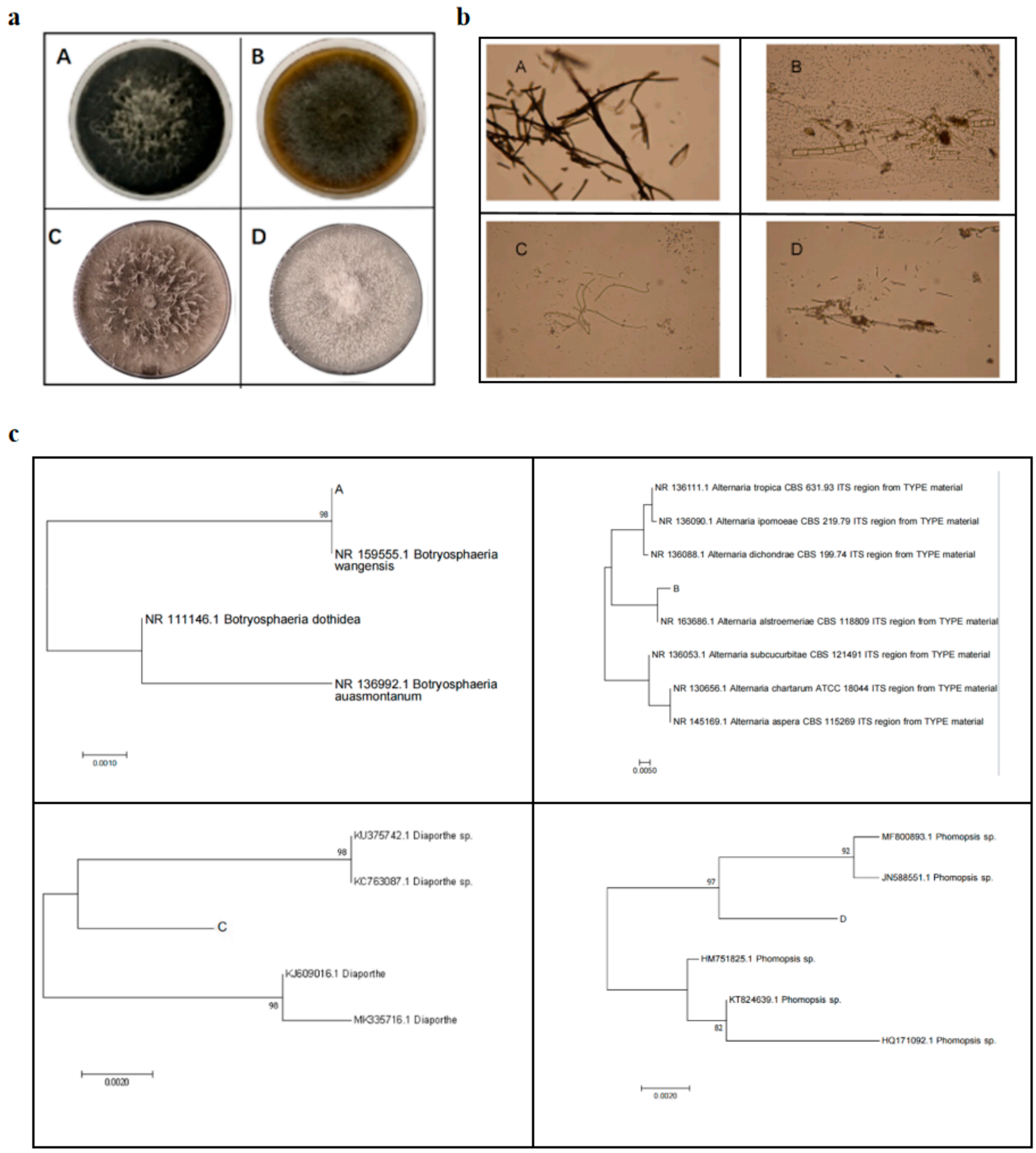

Figure. S1. Colony morphology (a), Morphology of conidiophore (b), and Phylogenetic tree (c) of isolates from kiwifruit: *Botryosphaeria dothidea* (A), *Alternaria* sp. (B), *Diaporthe* sp. (C), *Phomopsis* sp. (D).
