# Supplementary figures and images for: Pullulan-Based Active Coating Incorporating Potassium Metabisulfite Maintains Postharvest Quality and Induces Disease Resistance to Soft Rot in Kiwifruit
Source: Foods. 2023 Aug 24;12(17):3197. doi: 10.3390/foods12173197 (PMC10487164; doi:10.3390/foods12173197)

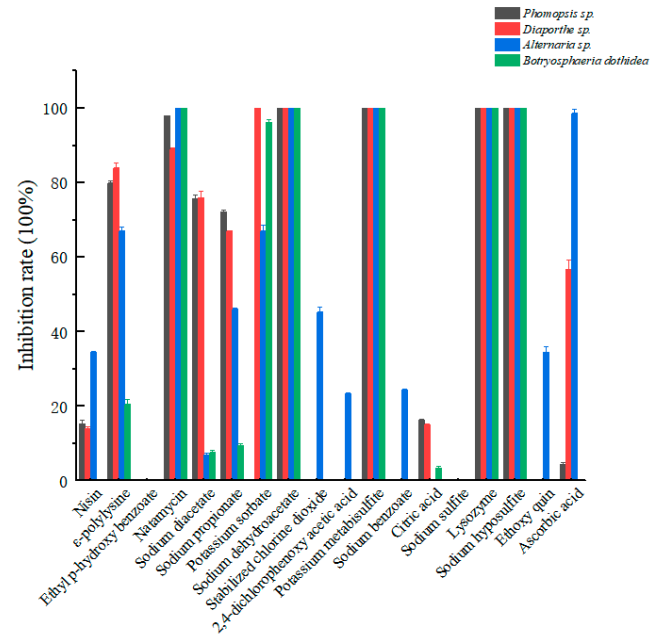

**Figure. S2.** Inhibition rate of 18 food additives on four pathogens.

Supplement: Supplementary file 1 [file foods-12-03197-s001.zip › Figure S2.pdf]
